# Diagnostic Methods for Anesthesia*Extract from an article printed in the December, 1915, *New Jersey Dental Journal*.

**Published:** 1916-02

**Authors:** Francis Ashley Faught

**Affiliations:** Philadelphia


### DIAGNOSTIC METHODS FOR ANESTHESIA.*

BY FRANCIS ASHLEY FAUGHT, M.D., OF PHILADELPHIA.

In considering the practical side of this subject, it appears that I can best deliver my message to you by outlining what, from my standpoint, should be the sum of knowledge possessed by the anesthetist—be he physician, dentist or nurse. The object of this knowledge, as already intimated, is to permit of a rapid and accurate separation ot all patients into two groups, namely, those to whom analgesia and anesthesia may be given without risk, other than that of the anesthetic itself; and those to whom the administration of anesthesia incurs unnecessary risk or in whom it results disastrously, either immediately or some time later.

Before the administration of any anesthetic, we should endeavor to determine the presence of disease by the methods of inspection, palpitation, percussion, ausculation, and by special investigations directed toward the circulation and the kidneys. An experienced diagnostician can in most cases reach a satisfactory conclusion by a well directed general inspection alone. The minority require additional methods.

The risk of anesthesia is increased in all those who are evidently in poor health, irrespective of its cause. This poor health may be due to chronic disease of the internal organs, or to the result of recent disease in patients who present themselves for dental operations too early in their convalescence. Thus extreme emaciation and physical weakness would suggest malignant disease, some form of Bright's disease, or pulmonary tuberculosis; whereas obesity, either accompanied by extreme pallor or flushing, is

* Extract from an article printed in the December, 1915, *New Jersey Dental Journal.*

suggestive of cardiovascular and heptic disorders. The addition of edema of the hands, ankles or face, cyanosis, physical weakness or dyspnea (shortness of breath) should augment our suspicions. A few questions relative to remote or recent acute or chronic illnesses will often explain the abnormal condition and will suggest the propriety of a short postponement to permit of more complete recovery.

Palpation serves to confirm some of the points brought out by inspection, thus, thickened and tortuous blood vessels, palpitation of the heart, and the presence of edema are determined by this means. By percussion we determine gross variations in pulmonary resonance, liver enlargement and alteration in the size of the heart. By auscultation, we determine changes in the respiratory and cardiac (heart) sounds, also the presence or absence of abnormal sounds within the lungs, as indicating bronchitis and other infections, and the presence or absence of heart murmers. Among the special methods of value are the study of the blood pressure and more rarely the examination of the urine.

The subject of physical diagnosis is too broad and the time too short to permit of full discussion, explanation or demonstration at this time, neither could such a demonstration be carried out successfully outside of the general hospital or the medical dispensary clinic.

If any one part of this work be possessed of greatest value and widest applicability I believe it to be the examination of the circulation, for hardly any condition of importance affecting the safety of anesthesia can exist in severe form or for a prolonged period without being reflected by changes in this system.

I believe that every dental practitioner should be able to recognize departures from normal conditions in the circulation and be able to determine the cases unsuitable for dental anesthesia. He should be familiar with variations in the pulse, the normal location of the apex-beat, the presence or absence of thrills and the presence of abnormal pulsations in the vessels of the neck. He should be able to determine the rate, rhythm and relative force of the pulse and have a speaking acquaintance with the common forms of heart murmur. He should also be able to make blood pressure observations and rationally employ this data before and during the administration of anesthetics.

It would be superfluous for me to discuss with you normal physiology and anatomy, because these studies were a part of your dental education and because this knowledge is easily acquired from modern text-books or physical diagnosis. A short course upon these subjects by a competent instructor will, if necessary, refresh your memories.

Concerning the blood pressure test, you are doubtless aware that the surgeon of to-day has come to look upon this procedure as a most important and signal aid, not only to determine the relative safety of the several anesthetics, but also as a guide to the condition of the patient during their administration. We are now in possession of sufficient experimental and clinical data to enable us to state quite definitely what changes in blood pressure may be expected before, during and after anesthesia, and to decide what are the natural changes to be expected from the operative procedures themselves, as compared with abnormal phenommena which demand attention.

We also know the effects of the various anesthetics upon the blood pressure. Thus during the first moments of the administration of the general anesthetics there is a primary rise in systolic pressure, which is the sum of mental excitement coincident to the procedure plus the stimulating effect of the drug. During the course of the administration of chloroform and ethyl-chloride and the mixed anesthetics, into which chloroform, or ethyl-chloride enter, we find that, promptly after the primary stage of elevation, there is a gradual fall in systolic pressure, the degree of which depends largely upon the concentration of the drug and the duration of its administration. With ether we have the usual primary rise which is speedily followed by a return to normal, at which level the pressure remains, even in administration of long duration, and with the employment of large quantities of the anesthetic. This fact makes ether the anesthetic of choice in all operations involving shock or in persons of reduced vitality. $N_2O$ alone is accompanied by a primary rise, followed by a temporary reduction which is again relieved by a sharp rise incident to the partial asphyxia which accompanies profound anesthesia by this drug. The administration of O plus $N_2O$ modifies to a degree both the primary and the secondary rise. This difference is due largely to the elimination of the asphyxial element present in pure $N_2O$ anesthesia.

Various operative procedures have been found to affect the blood pressure level, thus skin incisions or sensory nerve irritation, unless sufficiently intense to produce shock, are accompanied by a rise in systolic pressure. Respiratory obstruction with incidental partial asphyxia causes a sharp systolic rise which is transitory, disappearing promptly when the asphyxia is removed. The handling of large nerve trunks result in a temporary rise, unless this irritation is so profound as to produce shock. Any procedure which is recognized to rapidly cause shock is uniformly attended by a falling pressure, which may rapidly become dangerously low.

Concerning the normal limits of blood pressure and the indications arising from the discovery of abnormal pressures but few words are required. Thus high blood pressure, if discovered previous to anesthesia, and not explained on the grounds of a temporary rise due to mental excitement, is fairly conclusive evidence of some conditions involving the heart, arteries or kidneys, usually all three, and should be given due weight in determining against anesthesia unless urgently demanded. Abnormally low pressures in the absence of evident shock suggests the presence of some cachetic state, or a convalescent from some acute illness, as grippe or typhoid fever or the presence of active pulmonary tuberculosis.

While it may be stated that a normal preliminary blood pressure reading is the first requirement to safe administration of the anesthetic, nevertheless it cannot be said that the presence of an abnormal pressure is an absolute contra-indication of this procedure. It is here that judgment and experience largely enters, which can only be acquired by experience. I believe that I am safe in saying that when there is any doubt in the mind of the operator, it is advisable to call in a member of the medical profession to settle the question. Such a course will be appreciated by the intelligent patient, and is for your own protection.

